# Development and Validation of a Nomogram for Predicting the Unresolved Risk of Parents of Adolescents With Psychiatric Diagnoses

**DOI:** 10.3389/fpsyt.2022.796384

**Published:** 2022-04-01

**Authors:** Qingqing Sheng, Chunfeng Cai, Pingdong Li, Lihua Chen, Xi Zhang, Xinyu Wang, Yucui Gong

**Affiliations:** ^1^Guangzhou Institute of Respiratory Health, The First Affiliated Hospital of Guangzhou Medical University, Guangzhou, China; ^2^School of Health Sciences, Wuhan University, Wuhan, China; ^3^Nursing Department, The First Affiliated Hospital of Guangzhou Medical University, Guangzhou, China; ^4^The First Clinical Medical College, Anhui Medical University, Hefei, China

**Keywords:** psychiatric diagnoses, nomogram, parents, resolution, prediction model, unresolved risk

## Abstract

Evaluating the resolution of parents of ill children can help in taking measures to alleviate their distress in a timely manner and promote children's rehabilitation. This study aims to develop and validate a nomogram for predicting the unresolved risk of parents of adolescents with psychiatric diagnoses. The data for 130 parents (modeling dataset = 90; validation dataset = 40) were collected. A nomogram was first developed to predict the unresolved risk for parents based on the logistic regression analysis in the modeling dataset. The internal and external validation then were conducted through quantifying the performance of the nomogram with respect to discrimination and calibration, respectively, in the modeling and validation datasets. Finally, the clinical use was evaluated through decision curve analyses (DCA) in the overall dataset. In the results, the nomogram consisted of six risk factors and provided a good discrimination with areas under the curve of 0.920 (95% *CI*, 0.862–0.978) in internal validation and 0.886 (95% *CI*, 0.786–0.986) in external validation. The calibration with good consistency between the observed probability and predicted probability was also found in both internal and external validation. DCA showed that the nomogram had a good clinical utility. In conclusion, the proposed nomogram exhibited a favorable performance with regard to its predictive accuracy, discrimination capability, and clinical utility, and, thus, can be used as a convenient and reliable tool for predicting the unresolved risk of parents of children with psychiatric diagnoses.

## Introduction

The arrival of a new child into the family is usually received with immense elation, and parents generally have optimistic expectations of what their child will become and how they will be as parents (1, 2). Parents usually invest a large amount of energy and time in childrearing, while also enjoying their parenting role. When facing with children's diagnoses of an illness or a disability, parents can suffer from disappointment and distress because of failing to achieve their hopes of a healthy offspring or a “perfect child” (3). Moreover, the process of accepting or adjusting a disease diagnosis is most challenging for parents because changing previous expectations to more realistic ones can be as painful as losing a loved one (4–6).

Coming to fully accept a child's diagnosis requires parents to cognitively and emotionally process the experience of children with an illness or a disability. Cognitively, parents need to understand the implications of their child's diagnosis for themselves and their child. Emotionally, they need to acknowledge the feelings associated with the child's diagnosis and comprehend the meaning of parenting a child with a medical condition (7, 8). Parents who accepted their children's diagnoses were found to be more likely to build secure attachment with children, be more sensitive to children's cues, and better adjust their reactions to children, even when children's responses were inappropriate (9, 10). Studies have shown that parents' reactions to children's diagnoses were the premise of providing support for children, which had a major influence on the disease progress, treatment, and rehabilitation (11). Thus, professionals should be aware of the significance of assessing parents' reactions and identifying the challenges faced by parents in the process of adaptation (7, 12, 13).

An important progress in assessing parents' reactions to children's diagnoses has been made by Marvin and Pianta (10) with the introduction of the model of the resolution of the diagnosis based on the attachment theory. Resolution represents a process that parents change their working models of the anticipated perfect child into conceptions that are more aligned with the reality of child with specific abilities and limitations (14). Then a standardized, reliable procedure named the Reaction to Diagnosis Interview (RDI) was developed to identify individual differences in the process of the parents' resolution (8, 10, 15). The resolution status of parents was classified as two main classifications: “resolved” or “unresolved” according to RDI. Resolved parents can quickly cease active grieving and reorient current realities and future possibilities, which is characterized by acknowledging feelings associated with diagnoses, controlling their emotional responses, and moving on in life. On the contrary, unresolved parents display an absence of these characteristics. They are described as emotionally “stuck in the past” and tend to preoccupy themselves with negative aspects of their children's condition (10).

In recent years, studies have investigated the resolution of parents of children with various medical conditions, including autism spectrum disorder (13, 16), epilepsy (10), developmental delay (17), cerebral palsy (2, 10, 18), diabetes (19), preterm birth (20), and phenylketonuria (12). These literatures showed that the rates of “resolved” were diverse with a range between 33% (13) and 82% (2), supporting the view that the resolution was related to the illness itself. Except for the type of illness, some other demographic variables were also found to be associated with parents' resolution. An earlier study has shown that parents' stress and burden became greater as children's age progresses, making it more difficult for parents to resolve the children's diagnoses (17). The same was true as the parents got older. Some studies have revealed that fathers were more likely to resolve children's diagnoses than mothers, yet these differences did not reach significance (12, 21). Parents with lower education levels reported a heavier burden and higher unresolved status. Besides, family function and social support were also related to the parents' resolution (22, 23).

At present, parents' resolution to their children's psychiatric diagnosis did not receive a great deal of attention. Adolescence is the vital period for most people to pursue their education, seek for suitable job, and establish friendships and romantic relationships. Psychiatric disorder, often beginning at adolescence, cannot only potentially limit the achievement of these objects but also lead to high rates of disability, mortality, and suicide (24, 25). Hence, like parents of children with other medical conditions, parents of adolescents with psychiatric disorders may also experience strong emotional distress and have difficulties in resolving their children's diagnoses (26). Besides, obvious behavior problems, stigma, and stereotypes of psychiatric disorders may also prevent parents from resolving their children's diagnoses (11, 27). Therefore, identifying the high-risk unresolved parents and developing targeted interventions for them should become an integral part of the clinical care of adolescents with psychiatric diagnoses.

Nomogram is a statistics-based tool that can quickly predict the probability of a specific event through approximate complicated calculation without a computer or calculator (28, 29). As a simple and convenient mathematical model, nomograms have recently attracted increasing attention for their substantial clinical utility. This study aims to establish and validate a nomogram for predicting the unresolved risk of parents of adolescents with psychiatric diagnoses in an intuitive, visual, and convenient way.

## Methodology

### Overview of the Design

It was a cross-sectional study incorporating both interview and questionnaire with a sample of parents of adolescents with psychiatric diagnoses.

### Participants

A convenience sample was employed to recruit participants from a mental health center between November 2019 and February 2021 in China. The inclusion criteria for parents were (a) having a child (10–18 years old) meeting the diagnostic criteria (DSM-IV) for psychiatric disorder, (b) spending more time with their child than other family members, and (c) willing to be interviewed and share their experiences of caring for their child. Parents with a mental illness or lack of cognitive capacity were excluded. The total sample comprised 130 parents of adolescents with psychiatric diagnoses, and these parents were randomly divided into modeling dataset (90 cases) and validation dataset (40 cases) with a split ratio of 7:3 (30, 31).

### Procedures

Ethical approval was obtained from the Institutional Review Board of Wuhan University School of Medicine prior to the study. Research assistants spoke with parents who met the criteria about the goals, significance, and methods of the study, and asked if they and their child would like to participate. Upon parents' consent to participate, appointments were made for interviews in an unoccupied ward. All interviews were audio-recorded by a minirecording device. Notes were made on behavioral characteristics (i.e., body language and facial expression) during the interview in order that the parents' perspectives were correctly stated and comprehended. Appropriate pauses or changes in topic were applied whenever participants seemed to feel uncomfortable and upset during the interview. After completing of the RDI, parents were asked to independently complete the self-report questionnaire including the sociodemographic characteristics, the Family Adaptability and Cohesion Evaluation Scales (FACESII), the Parenting Stress Index—Short Form (PSI-SF), and the Social Support Rating Scale (SSRS). The researchers remained quiet when the patients filled out the questionnaire. All data were anonymous and only accessible to the researchers.

### Measures

#### Reaction to Diagnosis Interview

Reaction to Diagnosis Interview (RDI) is used to assess the resolution status of parents of children with medical conditions (15, 32). It is a standardized, videotaped, and structured interview consisting of five questions. The interview may last from a few minutes to approximately 15 min. In the interview, parents were asked (1) to recall the period of time they began noticing that their children had a medical problem; (2) how they felt at that time and if there were changes to those feelings; (3) to describe the events and experiences surrounding the time they received the diagnosis; (4) how their feelings changed since the time of the diagnosis; (5) to detail whether they have been searching for other reasons for their experiences. Each interview was videotaped, transcribed, and classified as “resolved” or “unresolved” (8). Reliability and validity of the RDI have been confirmed, with inter-rater agreement ranging from 88 to 96% (3, 15). The coding was completed by two trained coders, and each trained coder rated all interviews and was blinded to each other's ratings. Researchers had an intercoder agreement of 90% on the “resolved” or “unresolved” classification in the study.

#### Sociodemographic Characteristics

The questionnaire was designed based on the purpose of this study. It provided data on sociodemographic variables of the parents including age, gender, marital status, education level, and status of employment, as well as data of the children including age, gender, one child, and time since diagnosis.

#### Family Functioning

The family functioning is evaluated by the Family Adaptability and Cohesion Evaluation Scales (FACESII). The 30-item self-report scale consists of two subscales, namely, coherence (16 items) and adaptability (14 items). All items are scored based on a five-point Likert scale (ranging from 1 = almost never to 5 = almost always). A higher score indicates better family functioning. The validity and reliability of the FACESII of the Chinese version have been well established (33), and its internal consistency was acceptable (Cronbach's α = 0.73–0.85), and the test–retest reliability was also satisfactory (r = 0.84–0.91).

#### Parenting Stress

The Parenting Stress Index—Short Form (PSI-SF) (34) is a 36-item self-report scale designed to identify the experiencing stress of the parent–child system and the sources of that stress. The scale is organized into three subscales consisting of 12 items each: parental distress (PD), parent–child dysfunctional interaction (PCDI), and difficult child (DC) (34). All items are scored based on a five-point Likert scale (ranging from 1 = strongly agree to 5 = strongly disagree). A higher score indicates higher parenting stress. The PSI-SF has been translated into Chinese and previously used in the Chinese population, and has been tested to have good reliability and validity (35–37).

#### Social Support

Originally developed in Chinese by Xiao, the Social Support Rating Scale (SSRS) is a self-rated scale used to measure social support, with a good test–retest reliability of 0.92 (38). It has already been widely used in different Chinese communities and showed good validity and reliability (39–41). The 10-item scale contains three dimensions including objective support, subjective support, and support usage to evaluate social support. A higher score indicates higher social support.

### Decision Curve Analysis

The decision analysis curves (DCA) was introduced by Vickers and Elkin to estimate clinical utility of prediction models based on the threshold probability (probability that triggers a medical intervention, equating to the probability at which the harm of a false-positive intervention exceeds the harm of a false-negative non-intervention) (42). The threshold probability is used to derive the net benefit (defined as the fraction of true positives subtracted by the fraction of false positives weighted by the relative harm of a false-positive and false-negative result) (43). Graphical analysis of the net benefit and the threshold probability yields a decision analysis curve, which can then be used to assess the net benefit of nomogram-assisted decisions at different threshold probabilities, compared with the net benefit of decisions made with the assumption that either all or no patient has the outcome of interest (42–44).

### Data Analysis

All statistical analyses were conducted using R Environment for Statistical Computing (version 4.0.2, http://www.r-project.org/). Univariable and multivariable logistic regression analysis was performed to identify independent risk factors for the unresolved parents of adolescents with psychiatric diagnoses. All tests were two sided, and values of *p* < 0.05 were considered statistically significant. Then a nomogram was created using the “rms” package based on the independent risk factors of logistic regression analysis. The performance of the nomogram was evaluated through measuring discrimination and calibration in both the modeling and validation datasets. Discrimination was evaluated by using a receiver operating characteristic (ROC) curve and the area under the curve (AUC). The calibration was assessed by observing the goodness of fit between the observed probability and the predicted probability in the calibration plot. Finally, the decision curve analysis (DCA) was performed to evaluate the clinical utility of the nomogram based on net benefits at different threshold probabilities in the overall dataset (modeling and validation datasets).

## Result

### The Characteristics of Parents and Adolescents

A total of 150 parents of adolescents with mental illness were invited to participate in our study; 20 parents rejected to participate for the main reasons that they “do not want” and “have no time.” In the end, the study included 130 eligible parents, including 90 parents in the modeling dataset and 30 parents in the validation dataset. The characteristics of parents and adolescents in the modeling dataset, validation dataset, and overall dataset are shown in Table 1.

**Table 1:** Characteristics of parents and adolescents in the modeling, validation, and overall datasets.

**Characteristics**	**Overall dataset (*n* = 130)**	**Modeling dataset (*n* = 90)**	**Validation dataset (*n* = 40)**
**Resolution status**, *n* (%)			
Unresolved	63 (48.5)	45 (50.0)	18 (45.0)
Resolved	67 (51.5)	45 (50.0)	22 (55.0)
**Parental variables**			
Age (M ± SD)	39.68 ± 3.54	39.37 ± 3.33	40.38 ± 3.92
Gender, *n* (%)
Female	83 (63.8)	53 (58.9)	30 (75.0)
Male	47 (36.2)	37 (41.1)	10 (25.0)
Marital status, *n* (%)			
Married	114 (87.7)	78 (86.7)	36 (90.0)
Single	16 (12.3)	12 (13.3)	4 (10)
Education level, *n* (%)			
Bachelor's degree	42 (32.3)	26 (28.9)	16 (40.0)
High school	38 (26.2)	28 (31.1)	10 (25.0)
Middle school	42 (32.3)	30 (33.3)	12 (30.0)
Junior school	8 (6.2)	6 (6.7)	2 (5.0)
Status of employment, *n* (%)			
Yes	71 (54.6)	49 (54.5)	22 (55.0)
No	59 (45.4)	41 (45.6)	18 (45.0)
**Children variables**			
Age (M ± SD)	14.68 ± 2.07	14.77 ± 1.99	14.47 ± 2.26
Gender, *n* (%)			
Female	61 (46.9)	42 (46.7)	19 (47.5)
Male	69 (53.1)	48 (53.3)	21 (52.5)
One child, *n* (%)			
No	66 (50.8)	45 (50.0)	21 (52.5)
Yes	64 (49.2)	45 (50.0)	19 (47.5)
Time since diagnosis (month), *n* (%)			
>24	38 (29.2)	26 (28.9)	12 (30.0)
6–24	55 (42.3)	39 (43.3)	16 (40.0)
1–6	37 (28.5)	25 (27.8)	12 (30.0)
**Other variables**			
PSI (M ± SD)	103.75 ± 11.03	103.86 ± 11.06	103.50 ± 11.09
FACES (M ± SD)	94.03 ± 7.14	94.11 ± 7.55	93.85 ± 6.22
SSRS (M ± SD)	34.62 ± 4.59	34.74 ± 4.88	34.35 ± 3.88

*M, mean; SD, standard deviation; PSI, Parenting Stress Index; FACES, Family Adaptability and Cohesion Evaluation Scale; SSRS, Social Support Rating Scale*.

### The Logistic Regression Analysis in the Modeling Dataset

In the modeling dataset, the univariable and multivariable analysis showed that parents' gender, status of employment, adolescents' age, one child, time since diagnosis, and social support were the independent risk factors related to the unresolved risk of parents of adolescents with psychiatric diagnoses (see Table 2).

**Table 2:** Risk factors for unresolved status by univariable and multivariable logistic regression analysis in the modeling dataset.

**Characteristics**	**Univariable** **analysis**		**Multivariable** **analysis**	
	**OR (95% CI)**	***p***	**OR (95% CI)**	***p***
**Parental variables**				
Age	0.883 (0.770–1.004)	0.065	–	–
Gender				
Female	Reference		Reference	
Male	0.627 (0.446–0.724)	<0.001	0.473 (0.231–0.638)	0.018
Marital status				
Married	Reference		–	–
Single	1.596 (0.609–2.135)	0.672	–	–
Education level				
Bachelor's degree	Reference		Reference	
High school	1.531 (0.565–2.075)	0.421	2.342 (0.634–3.078)	0.803
Middle school	7.750 (0.812–9.051)	0.108	3.840 (0.167–4.506)	0.275
Junior school	4.032 (3.125–5.303)	0.016	2.801 (0.193–6.178)	0.731
Status of employment				
Yes	Reference		Reference	
No	2.984 (1.242–4.342)	0.023	2.494 (1.259–3.485)	0.012
**Children variables**				
Age	1.627 (1.446–1.824)	<0.001	1.415 (1.263–1.648)	0.005
Gender				
Female	Reference		–	–
Male	1.139 (0.998–1.301)	0.054	–	–
One child				
No	Reference		Reference	
Yes	8.500 (6.294–9.935)	<0.001	7.541 (5.353–9.412)	0.017
Time since diagnosis (month)				
>24	Reference		Reference	
6–24	3.322 (2.102–4.283)	0.019	2.604 (0.215–3.107)	0.342
1–6	4.854 (3.163–5.476)	0.004	3.521 (2.161–4.330)	0.015
**Other variables**				
PSI	1.918 (1.874–2.080)	<0.001	1.119 (0.949–2.365)	0.488
FACES	0.861 (0.795–0.933)	<0.001	0.667 (0.382–3.051)	0.733
SSRS	0.420 (0.237–0.672)	<0.001	0.746 (0.650–0.918)	0.015

*OR, odds ratio; PSI, Parenting Stress Index; FACES, Family Adaptability and Cohesion Evaluation Scale; SSRS, Social Support Rating Scale*.

### The Development and Internal Validation of Nomogram

**Figure 1:**
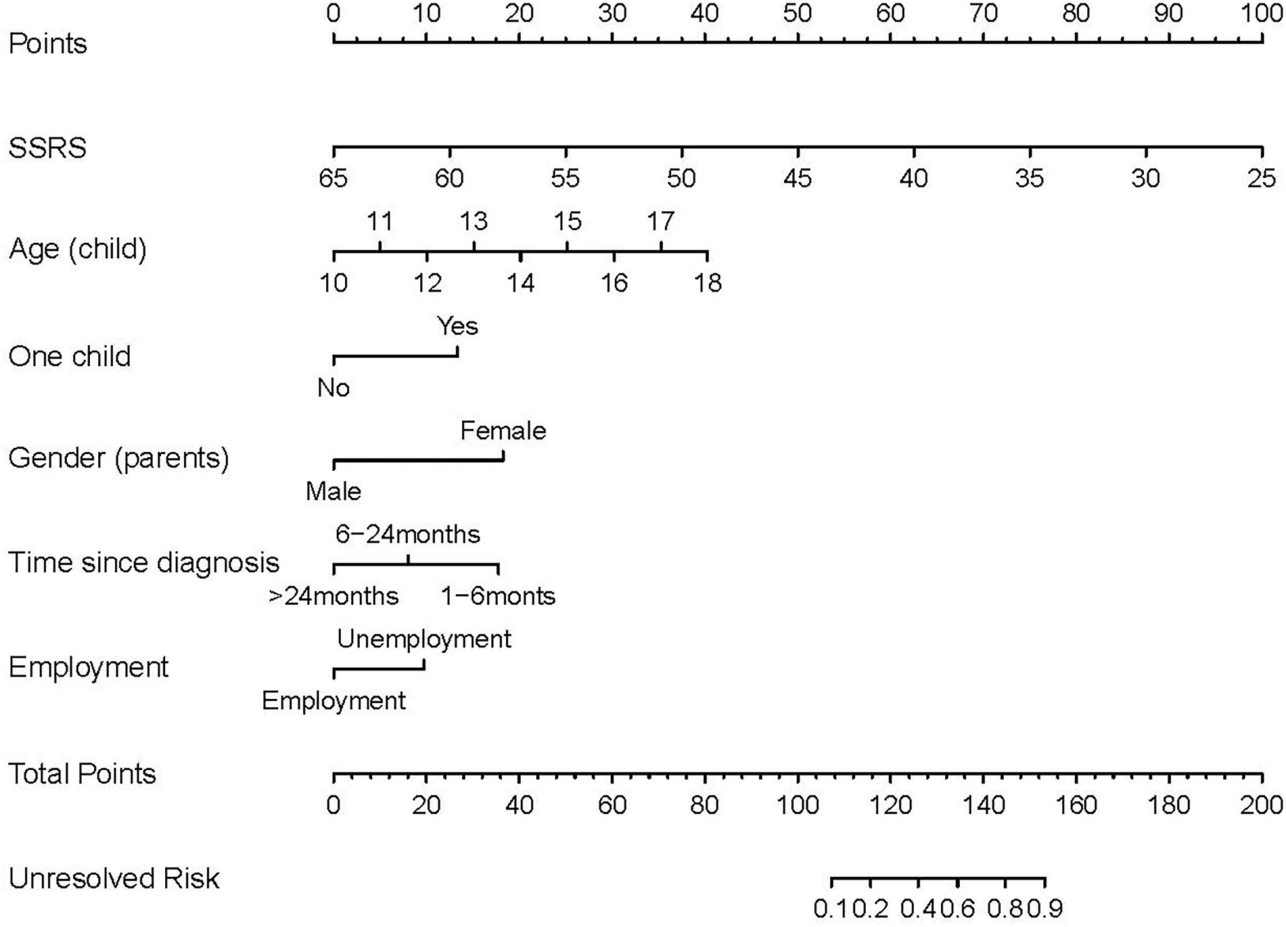
Nomogram for predicting the unresolved risk of parents of children with psychiatric diagnoses. Each risk factor of the parents has a value on its variable axis, and vertical lines are drawn upward to the point axis to obtain the corresponding scores for the value of each risk factor. A total score can be easily calculated by adding each single score. The total score is plotted on the bottom total point axis, and a vertical line is drawn downward to the unresolved risk axis to estimate the unresolved risk.

**Figure 2:**
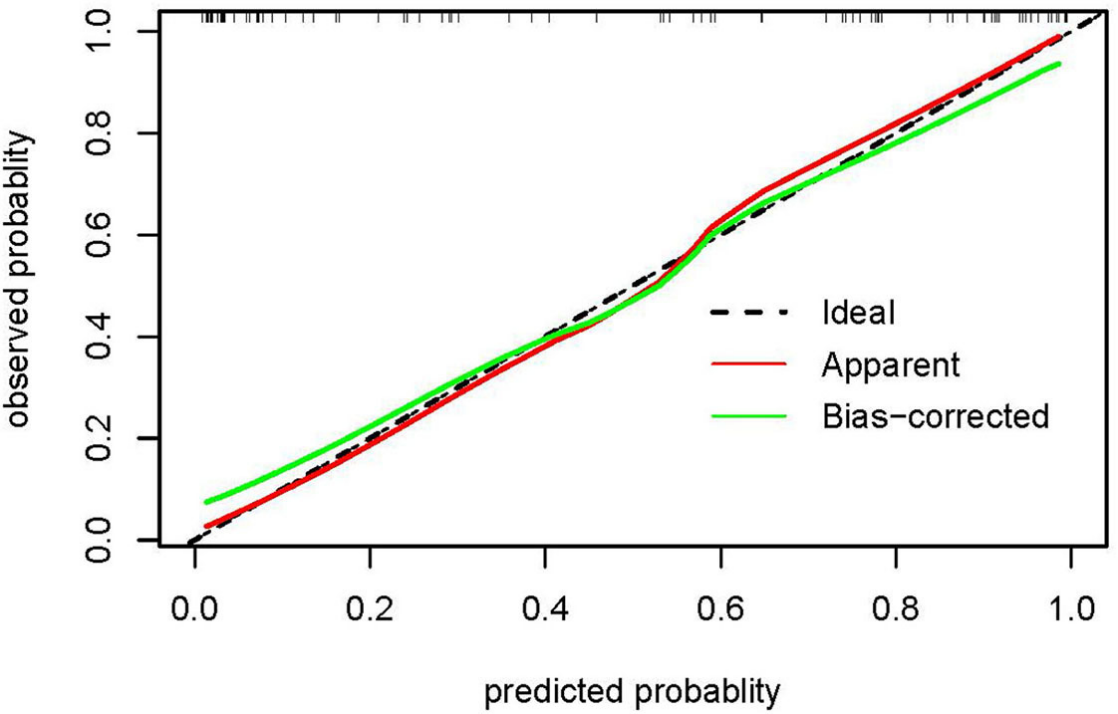
The calibration plot of the nomogram in the modeling dataset. The x-axis shows the nomogram-predicted probability of unresolved risk, and the y-axis shows the actual observed probability of unresolved risk. The ideal line (45°) in the calibration plots represents perfect consistency between the nomogram-predicted probabilities and the observed probabilities. The apparent line represents the model calibrated with the modeling dataset, and the bias-corrected line represents the model calibrated using the bootstrap method.

**Figure 3:**
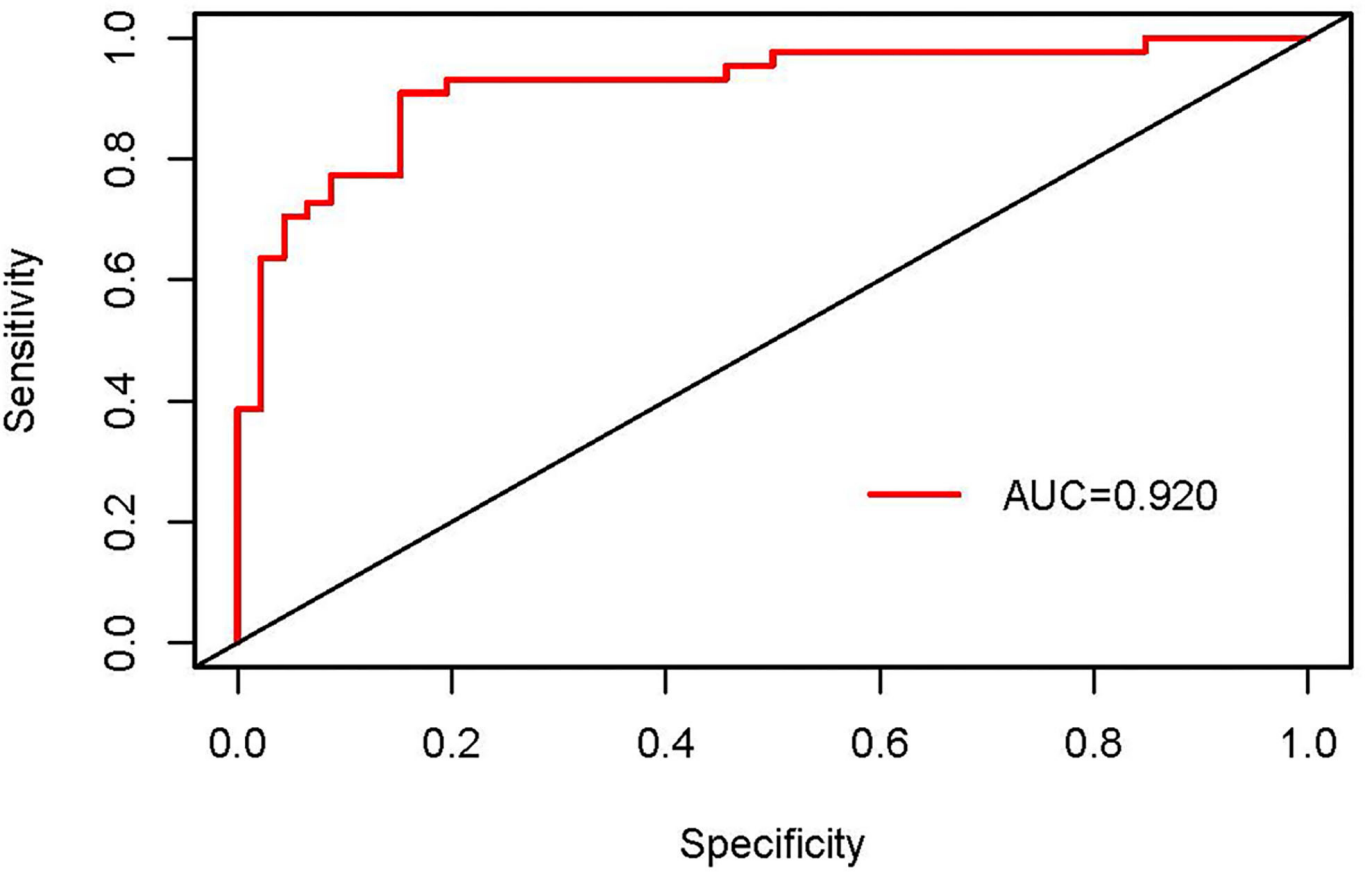
The receiver operating characteristic (ROC) of the nomogram in the modeling dataset.

Based on the significant variables in the logistic regression analysis, a nomogram for predicting the unresolved risk of parents of adolescents with the psychiatric diagnoses was developed in the modeling dataset (see Figure 1). The internal validation was conducted in the modeling dataset to assess the performance of the nomogram including calibration and discrimination. The calibration plot of the nomogram showed a good consistency between the predicted probability and observed probability (see Figure 2). The receiver operating characteristic (ROC) and the area under the curve (AUC) was 0.920, indicating favorable discrimination (see Figure 3).

### External Validation of Nomogram

**Figure 4:**
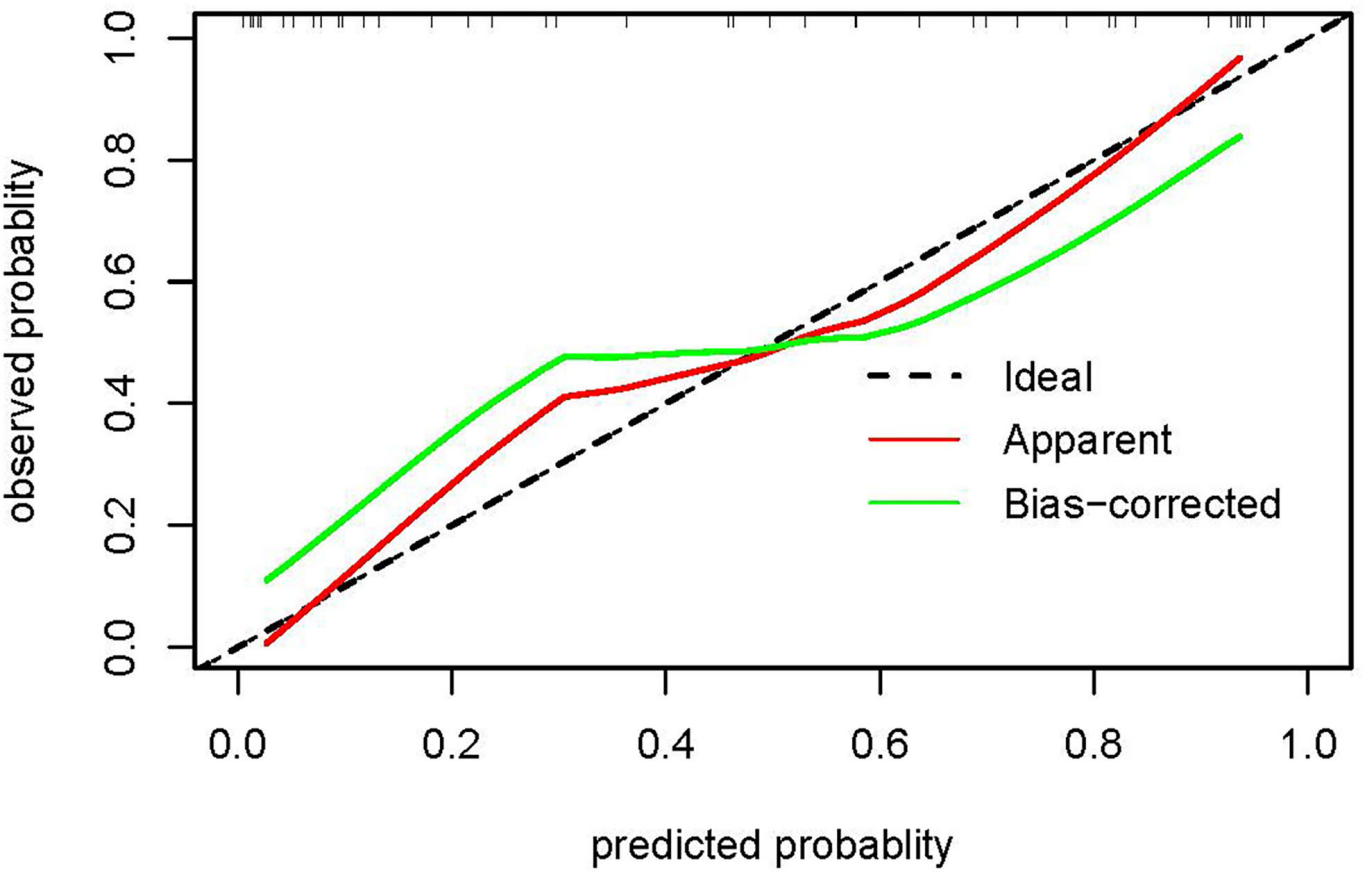
The calibration plot of the nomogram in the validation dataset. The x-axis shows the nomogram-predicted probability of unresolved risk, and the y-axis shows the actual observed probability of unresolved risk. The ideal line (45°) in the calibration plots represents perfect consistency between the nomogram-predicted probabilities and the observed probabilities. The apparent curve represents the model calibrated with the validation dataset, and the bias-corrected curve represents the model calibrated using the bootstrap method.

**Figure 5:**
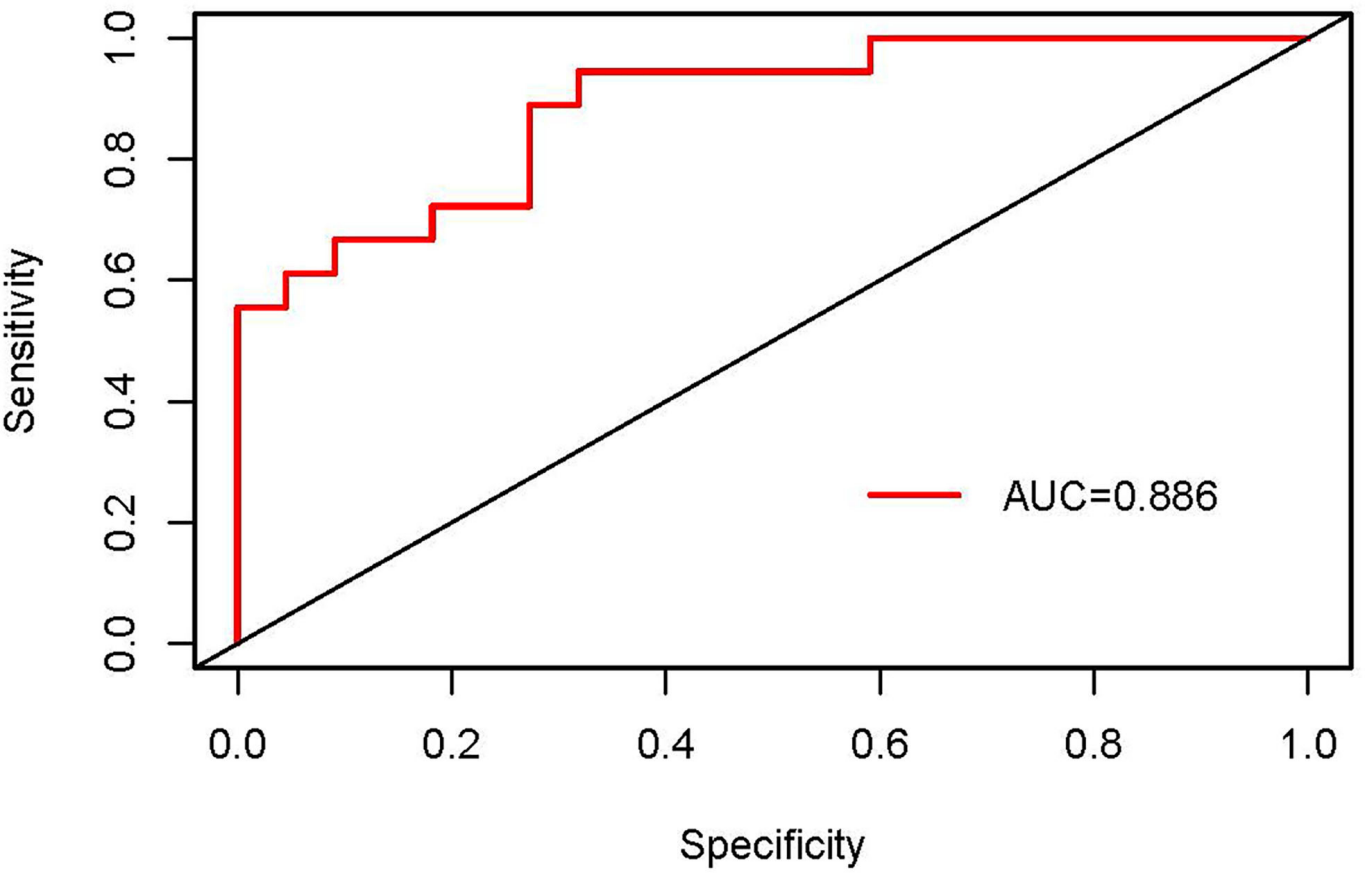
The receiver operating characteristic (ROC) curves of the nomogram in the validation dataset.

The validation of the nomogram was performed in the validation dataset to further evaluate the performance of the proposed nomogram. The calibration plot for the nomogram showed a good consistency between the predicted probability and observed probability (see Figure 4). The receiver operating characteristic (ROC) and the area under the curve (AUC) was 0.886, indicating good discrimination (see Figure 5).

### Clinical Utility of Nomogram

**Figure 6:**
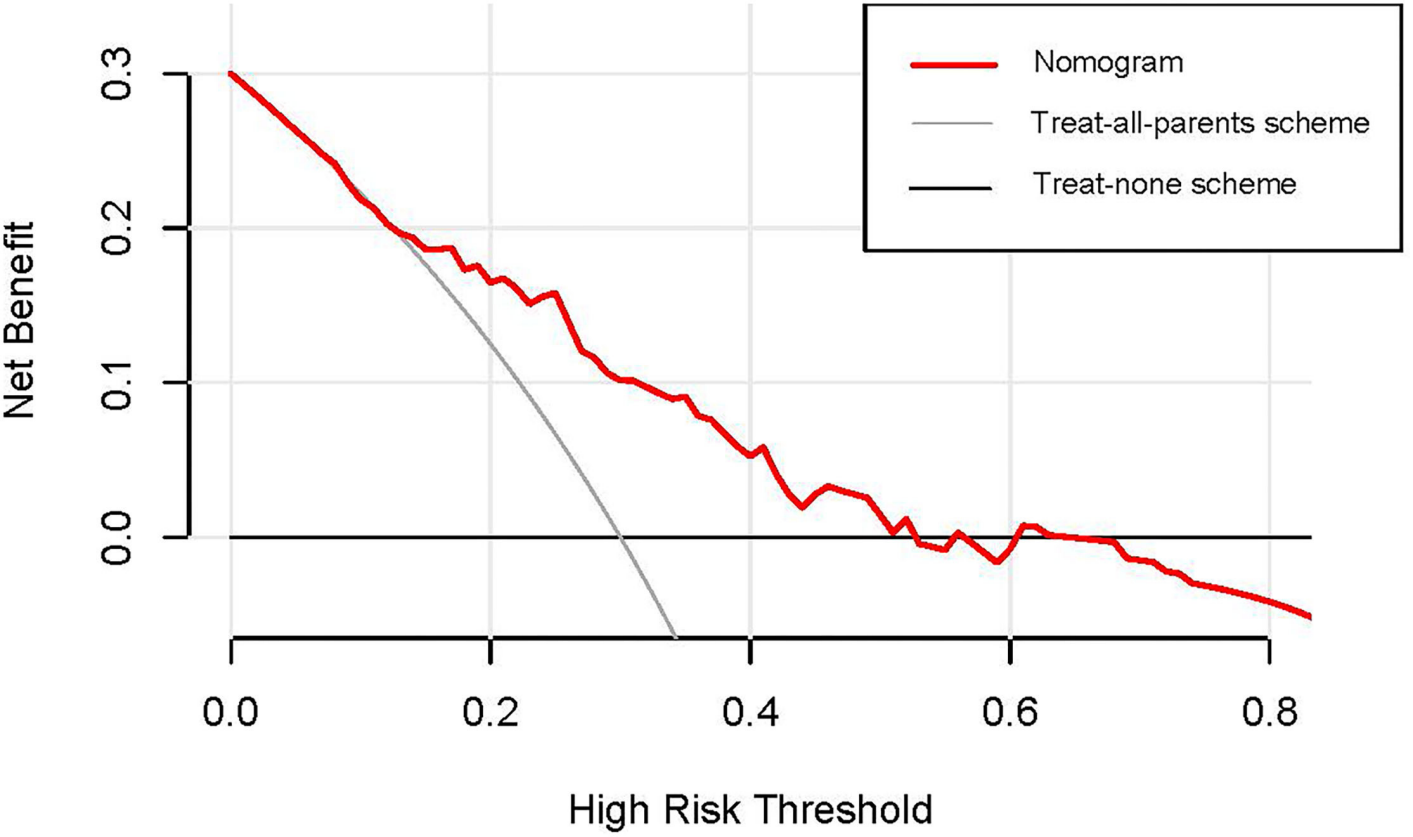
The decision curve analysis of the nomogram in the entire dataset. The y-axis measures the net benefit, and the x-axis measures the probability threshold. The red line represents the nomogram. The black line (treat-none scheme line) represents the assumption that all parents are unresolved statuses. The gray line (treat-all-parents scheme line) represents the assumption that no parent is unresolved status. The decision curve analysis (DCA) indicated that the nomogram provided more benefits than the treat-all-parents scheme and treat-none scheme when the threshold probability of unresolved status was >13% and <54%.

The decision curve analyses (DCA) was used to evaluate the clinical utility of the nomogram in all 130 parents. As shown in Figure 6, the nomogram provided greater benefits than the treat-all-parents scheme and treat-none scheme when the threshold probability was >13% and <54%. Therefore, the nomogram had a good clinical utility.

## Discussion

In the current study, the percentage of unresolved parents (50%) in the modeling dataset was higher than the mean percentage (40%) found in a previous study (45), which indicated that parents of adolescents with psychiatric diagnoses had higher rates of unresolved status. Therefore, evaluating the unresolved risk of parents in the process of resolution and making targeted interventions are vital in clinical care. In the current study, a nomogram was developed to predict the unresolved risk of parents of adolescents with psychiatric diagnoses based on the logistic regression analysis. It showed favorable discrimination, calibration, and clinical utility through internal and external validation. The proposed nomogram can be used as a tool to help professionals identify high-risk unresolved parents and take targeted interventions to promote parents' resolution of diagnoses.

Improving the accuracy of a model needs comprehensive and reasonable evaluation of all possible related variables, which is complicated and tedious work (46). In addition, it is hard to keep a balance between comprehensiveness and comprehensibility when creating the nomogram (46). Given the busy and complex clinical work, we, thus, only collected variables with clinical importance and easy accessibility to develop an easy-to-use prediction model. Based on the logistic regression analysis, we finally identified six independent risk factors including parents' gender, employment status, children's ages, one child, time since diagnosis, and social support, and developed the nomogram.

Consistent with previous studies (12, 17, 21), it was found that the mother had a higher unresolved risk than the father in our study. The gender roles theory indicates that women prefer to cope with the problem with personal emotion, and men are more stoic and logically driven. Therefore, mothers had more intense negative feelings than fathers in the interview and, thus, were more likely to be classified as an unresolved status. Employment status induced a positive effect for parents to resolve the children's psychiatric diagnoses in the study. It enabled parents to obtain stable financial resources and better medical resources, which can reduce parents' stress and promote the resolution of diagnoses (3). Parents of adolescents with shorter time since diagnosis had higher unsolved risk. It can be explained by Blacher's phase theory (47) describing that parents' reactions to their children's diagnoses gradually progress and finally achieve adaptation. As the only child in the family, the one child usually receives more attention from parents (48). Therefore, parents are more likely to immerse in distress and have difficulties in resolving the diagnosis. Moreover, caring for children with mental illness can be a challenging experience because of the special nature of the psychiatric disorder, such as obvious behavior problem, unclear prognosis, and lack of a radical cure. Formal and informal support can help parents to alleviate distress and stress, and, thus, are beneficial to the process of resolution (49). As for the children' ages, older children had more pronounced differences in abilities of independent living and communication than typical developing children, and thus, parents had more difficulties in resolving the diagnoses.

Validating the nomogram is essential to ensure that it can be applied generally and avoid overfitting. In the internal validation, the nomogram provided favorable discrimination with an AUC of 0.920 and a good calibration with goodness of fit between the predicted probability and observed probability. Research has indicated that the performance of a nomogram, such as discrimination and calibration, can vary when applied to different datasets (43). As a result, the external validation, validating in a dataset with similar characteristics to the population to which the nomogram will be applied, is generally regarded as the gold standard. In the current study, the external validation showed reasonable discrimination based on an AUC of 0.886 and a favorable calibration based on acceptable goodness of fit between the predicted probability and observed probability in the calibration plot. Although the performance of the nomogram in the validation dataset was slightly worse than those in the modeling dataset, the degraded performance metrics were still in the clinically acceptable range (43). Therefore, it was appropriate to recommend the nomogram for routine use in clinical practice.

However, even if the nomogram had a perfect performance, it was possible to lack clinical utility. The essential criterion to evaluate the utility of a model is whether the model benefits patients. When the threshold probability of the net benefit is impractical, the model with good performance may also have limited utility (46, 50). The decision analysis curve (DCA) was used to estimate the clinical utility of the nomogram based on the net benefit and the threshold probability in the present study. The results indicated that when the threshold probability of unresolved risk was 13%−54%, intervention for parents added more benefit compared with the treat-all-parents scheme or the treat-none scheme. Therefore, the nomogram can assist in decision making and have good clinical utility (51).

Kearney (26) designed the logistic prediction model for assessing the resolution of parents of young children with psychiatric disorders based on the data of 33 parents, but the model showed worse prediction ability for unresolved parents, indicating a need for further model development. Compared with the abovementioned logistic prediction model, the proposed nomogram in the current study showed some strengths. At first, the study expanded the resolution of the diagnosis to the adolescent psychiatric population and first established the nomogram for predicting the unresolved risk of parents of adolescents with psychiatric diagnoses in the context of the Chinese culture. Second, the nomogram had good calibration, discrimination, and clinical utility through internal and external validation. Third, the majority of variables identified by the nomogram were easily obtained and can reflect the clinically relevant information, which helped the medical staff to quickly identify the parents at high unresolved risk. Finally, this nomogram had a good cultural representation because it was created based on the Chinese population.

## Limitations and Future Direction

The limitations of this study should be acknowledged. First, selection bias was an issue in the study because there was no systematic sampling method, i.e., anyone who met the inclusion criteria was invited to participate. Second, the small size of the sample impacted the power of the analysis and the effect size, which was directly linked with the predictive ability of the model in logistic regression. Finally, participants were from a single institution, and the generalization of the nomogram to the other cities in China, or even the global population, was still unclear. Therefore, it is necessary for future studies to further explore the risk factors related to the unresolved status of parents and develop a model with universal applicability in the future. Regardless of the limitation of the nomogram, the study offered new perspective and research foundation for evaluating the parents' reactions to psychiatric diagnosis of their children.

## Conclusion

To our knowledge, we developed the first nomogram for predicting the unresolved risk of parents of adolescents with psychiatric diagnoses based on the routine medical information of parents and their adolescents. The proposed nomogram showed a favorable performance in predictive accuracy, discrimination, capability, and clinical utility. As a result, the nomogram can be considered as a prediction tool to guide medical staff to identify the high-risk unresolved parents and take personalized measures to assist them to resolve the diagnosis.

## Data Availability Statement

The raw data supporting the conclusions of this article will be made available by the authors, without undue reservation.

## Ethics Statement

Institutional Review Board of the Researchers' Institution granted approval for this study. Written informed consent was obtained from all participants.

## Author Contributions

QS, CC, and PL designed the study and wrote the research protocol. QS, CC, XZ, XW, and PL performed the literature, quality control, and collected and checked the data. QS, LC, YG, and PL analyzed the data, interpretation of data, and contributed to the in-depth revision of the manuscript. QS, PL, LC, YG, and XW prepared the manuscript. All authors contributed to and approved the final manuscript.

## Conflict of Interest

The authors declare that the research was conducted in the absence of any commercial or financial relationships that could be construed as a potential conflict of interest.

## Publisher's Note

All claims expressed in this article are solely those of the authors and do not necessarily represent those of their affiliated organizations, or those of the publisher, the editors and the reviewers. Any product that may be evaluated in this article, or claim that may be made by its manufacturer, is not guaranteed or endorsed by the publisher.
